# Cross-Validation Approaches for Replicability in Psychology

**DOI:** 10.3389/fpsyg.2018.01117

**Published:** 2018-07-02

**Authors:** Atesh Koul, Cristina Becchio, Andrea Cavallo

**Affiliations:** ^1^C'MON, Cognition, Motion and Neuroscience Unit, Fondazione Istituto Italiano di Tecnologia, Genova, Italy; ^2^Department of Psychology,University of Torino, Torino, Italy

**Keywords:** replicability, cross-validation, conceptual replication, direct replication, simulated replication

## Introduction

The ability to replicate a scientific discovery or finding is one of the features that distinguishes science from non-science and pseudo-science (Dunlap, 1925; Popper, 1959; Collins, 1985). In the words of Popper (1959):

“only by [such] repetitions can we convince ourselves that we are not dealing with a mere isolated ‘coincidence,' but with events which, on account of their regularity and reproducibility, are, in principle, intersubjectively testable.”

Recent years have seen a rising concern over the reproducibility of psychological science (Pashler and Harris, 2012; Doris, 2015; Open Science Collaboration, 2015), with some authors even claiming that most of the published research findings are irreproducible (Ioannidis, 2005). A systematic study designed to assess the reproducibility of psychological science (Open Science Collaboration, 2015) found that the mean effect size of the replicated studies was half of that of the originally conducted studies, and, even more strikingly, only 36% of replication studies had statistically significant results. To counter the concerns generated by these results and improve the quality and credibility of the psychological literature, two sets of strategies have been identified: (i) improvement in research methodologies; (ii) promotion of replication attempts.

Accumulating evidence is the scientific community's method of self-correction. When available, this is certainly the best available option for improving the scientific process. Unfortunately, replication attempts are not always feasible. For example, they may not be feasible for large clinical-epidemiological studies. Also, replicating studies that test extremely rare events or conditions (e.g., neuropsychological conditions) may be difficult or simply impossible. Therefore, the question arises as to the generalizability of replication attempts. Are there measures that one could adopt in such cases to ensure a high level of reproducibility despite the impossibility of reproducing the original study?

In this opinion article, we propose the incorporation of cross-validation techniques in single research studies as a strategy to address this issue. The first section of the article (section Replication) describes in brief what counts as a replication. In section Simulating Replicability via Cross-Validation Techniques, we introduce the concept of cross-validation and how this technique can be utilized for establishing replicability. Next, we describe different cross-validation schemes (section Cross-Validation Schemes), and finally, we conclude by highlighting some of the platforms for implementing cross-validation (section How to Apply Cross-Validation) and certain limitations (section Conclusions).

## Replication

Some controversies surrounding replication attempts seem to arise from the very notion of “replication”—the purposes of the attempt and when would it count as being successful (Simonsohn, 2013; Verhagen and Wagenmakers, 2014). Accordingly, multiple propositions of scope, functioning and typology have been identified (Lykken, 1968; Schmidt, 2009; Hüffmeier et al., 2016; Schmidt and Oh, 2016; LeBel et al., 2017).

A basic distinction is between direct replication and conceptual replication attempts (Zwaan et al., 2017). Direct replications are replication attempts that aim at reproducing the exact effects as obtained by a previous study, incorporating the exact experimental conditions. In contrast, conceptual replications examine the general nature of the previously obtained effects, while aiming at extending the original effects to a new context. For instance, a conceptual replication of an effect originally found in a monolingual population would attempt to replicate the effect in a bilingual population (e.g., influence of multiple language use on a particular phenomenon).

This article suggests a third typology of replication: *simulated replication*. When due to practical or methodological constraints direct replication and conceptual replication are not feasible or difficult to perform, simulated replication—we contend—provides an alternative approach to put the replicability of research findings to the test.

## Simulating replicability via cross-validation techniques

Simulated replicability can be implemented via procedures that repeatedly partition collected data so as to simulate replication attempts. Formally, this is referred to as *cross-validation*. Cross-validation entails a set of techniques that partition the dataset and repeatedly generate models and test their future predictive power (Browne, 2000). The partitioning can be performed in multiple different ways. The general format is that of a “leave k-observations-out” analysis. In such an analysis, the entire dataset is typically divided into k smaller observations (e.g., trials or participants). k-1 observations (training dataset) are used to generate and train a model. The validity and generalizability of the generated model is then tested on the k^th^ observation (test dataset).

Cross-validation has the computational advantage that it avoids fitting a model too closely to the peculiarities of a data set (overfitting). Overfitting can occur if a too-complex model is fitted to the training set. As an extreme example, if the number of parameters of a model is the same as or greater than the number of observations, then a model can perfectly predict the training data simply by memorizing the data in its entirety. Such a model, though, will typically fail severely when making predictions. Cross-validation avoids this risk by evaluating the performance of the model on an independent dataset (testing set). This protects against overfitting the training data. At the same time, it increases the confidence that the effects obtained in a specific study will be replicated, instantiating a simulated replication of the original study. In this way, cross-validation mimics the advantages of an independent replication with the same amount of collected data (Yarkoni and Westfall, 2017).

## Cross-validation schemes

Splitting data into training and test subsets can be done using various methods, including holdout cross-validation, k-fold cross-validation, leave-one-subject-out cross-validation, and leave-one-trial-out cross-validation. The choice of the most appropriate method depends on multiple factors including the sample size and the experimental design, the research question and the application (Gong, 1986; Borra and Di Ciaccio, 2010; Saeb et al., 2017; Varoquaux et al., 2017). This section provides a brief description of the most commonly used procedures.

### Holdout cross-validation

Holdout (or validation) is the simplest form of cross-validation, often considered as a validation method. Contrary to other methods, it splits the provided dataset only once into a training dataset and a testing dataset. The portion of data used for the training dataset is randomly selected, and the remaining part of the data, generally a fraction of 1/3 of the data, is assigned to the testing dataset. For instance, if collected data consists of 1,000 observations, a training set would typically consist of 667 observations, while 333 observations would be kept aside as a testing dataset for verifying the generated model. The advantage of this method is the lower computational load. However, its evaluation can have a high variance, depending on which data points end up in the training set and which end up in the test set.

### K-fold cross-validation

One way to improve over the holdout method is to divide the collected data into a certain number of equally sized observations or folds (k). One fold out of these (k) folds is chosen as testing dataset while the others (k-1) are used for the training purposes. This procedure is repeated k-times, each time selecting a different fold for testing purposes and the other folds (k-1) as training dataset. Consequently, k different accuracies are produced from the procedure. The variance of the resulting estimate is reduced as k is increased. A value of k = 10 is generally used as a rule of thumb for the number of folds. This method avoids the randomness emanating from estimates produced by splitting the data only once. The disadvantage is that the training algorithm has to be rerun from scratch k times, which means it takes k times as much computation to make an evaluation.

### Leave-one-subject-out cross-validation

Similar to k-fold cross-validation, the leave-one-subject-out approach repeatedly splits the data but instead of creating k-folds, the dataset is split according to the number of subjects in the dataset. Further, one subject is randomly selected for the testing purposes while the other subjects are used for training the model. This procedure is repeated until all the subjects have been used as test dataset. This method mirrors the clinically relevant use-case scenario of diagnosis and is therefore especially useful in clinical diagnostic applications, where the model is used to predict the clinical status of new individuals (Saeb et al., 2017).

### Leave-one-trial-out cross-validation

In the leave-one-trial-out approach k-fold cross validation is taken to its logical extreme, with k equal to N, the number of dataset samples. That is, one observation from the dataset is retained for testing and the rest of the observations are used to generate the model. This sampling is repeated until all the samples have been used as a testing data point. As before the average error is computed and used to evaluate the model.

## How to apply cross-validation

Implementations of cross-validation are a part of various packages such as Scikit-learn (Pedregosa et al., 2011), Pylearn2 (Goodfellow et al., 2013), PyMVPA (Hanke et al., 2009), Statistics and Machine learning toolbox (Matlab; MathWorks, Natick, MA), e1071 (Meyer et al., 2017), caret (Kuhn, 2008), and Microsoft Azure (Microsoft Corporation) among many others. These packages provide efficient implementations of state-of-the-art algorithms, reusable across scientific disciplines and application fields. The popular package Scikit-learn, for example, takes advantage of Python interactivity and modularity to assemble new models and programming new scripts to support different types of data from electroencephalography to functional magnetic resonance. While this flexibly expands possibilities for application, it also increases the required programming and computational skills. An important direction for research is the development of software that does not require advanced programming skills (e.g., PRoNTO http://www.mlnl.cs.ucl.ac.uk/pronto/; Schrouff et al., 2013). One of such software packages specifically developed to support psychologists is available from our group and is called “PredPsych” (Koul et al., 2017). This toolbox is endowed with multiple functionalities for multivariate analyses of data accompanied by guided illustrations on their implementation. Using this toolbox, researchers can employ various cross-validation schemes. By default, the syntax performs a k-fold cross-validation (i.e., *Results* = *classifyFun(Data,condition*). Alternative cross-validation methods (Holdout cross-validation, Leave-one-subject-out cross-validation, Leave-one-trial-out cross-validation) can be specified by simply supplying an additional argument to the basic cross-validation function (e.g., *Results* = *classifyFun(Data,condition,cvType* = “*holdout”*).

## Conclusions

In the current opinion article, we propose simulated replication using cross-validation as a way to mitigate the crisis in replication of effects in psychological science. Simulated replication is of course no panacea and should not be seen as a substitute for direct and conceptual replications. For instance, application of cross-validation techniques requires medium to large sample sizes as well as increased computational requirements. Simulated replications should thus, be rather considered as tool to improve confidence that the effects obtained in a single study would be replicated. We believe that the systematic adoption of this approach could help making replication a routine aspect of psychological science.

## Author contributions

AK and AC drafted the manuscript. CB provided critical revisions. All Authors approved the final version of the manuscript for submission.

### Conflict of interest statement

The authors declare that the research was conducted in the absence of any commercial or financial relationships that could be construed as a potential conflict of interest.
